# Factors associated with low bone mass in the hemodialysis patients – a cross-sectional correlation study

**DOI:** 10.1186/1471-2474-10-60

**Published:** 2009-06-04

**Authors:** Guey-Shiun Huang, Tzong-Shinn Chu, Meei-Fang Lou, Shiow-Li Hwang, Rong-Sen Yang

**Affiliations:** 1 Department of Nursing, College of Medicine, National Taiwan University, Taipei, Taiwan; 2 Department of Nursing, National Taiwan University Hospital, Taipei, Taiwan; 3 Department of Primary Care Medicine, College of Medicine, National Taiwan University, Taipei, Taiwan; 4 Department of Internal Medicine, National Taiwan University Hospital, Taipei, Taiwan; 5 Department of Orthopedics, College of Medicine, National Taiwan University, Taipei, Taiwan; 6 Department of Orthopedic Surgery, National Taiwan University Hospital, Taipei, Taiwan

## Abstract

**Background:**

Low bone mass is common in end-stage renal disease patients, especially those undergoing hemodialysis. It can lead to serious bone health problems such as fragility fractures. The purpose of this study is to investigate the risk factors of low bone mass in the hemodialysis patients.

**Methods:**

Sixty-three subjects on hemodialysis for at least 6 months were recruited from a single center for this cross-sectional study. We collected data by questionnaire survey and medical records review. All subjects underwent a bone mineral density (BMD) assay with dual-energy x-ray absorptiometry at the lumbar spine and right hip. Data were statistically analyzed by means of descriptive analysis, independent t test and one way analysis of variance for continuous variables, Pearson product-moment correlation to explore the correlated factors of BMD, and stepwise multiple linear regression to identify the predictors of low bone mass.

**Results:**

Using WHO criteria as a cutoff point, fifty-one subjects (81%) had a T-score lower than -1, of them 8 subjects (13%) had osteoporosis with the femoral neck most commonly affected. Regarding risk factors, age, serum alkaline phosphatase (ALP) level, and intact parathyroid hormone (iPTH) level had significant negative correlations with the femoral neck and lumbar spine BMD. On the other hand, serum albumin level, effective exercise time, and body weight (BW) had significant positive correlations with the femoral neck and lumbar spine BMD. Age, effective exercise time, and serum albumin level significantly predicted the femoral neck BMD (R^2 ^× 0.25), whereas BW and the ALP level significantly predicted the lumbar spine BMD (R^2 ^× 0.20).

**Conclusion:**

This study showed that advanced age, low BW, low serum albumin level, and high ALP and iPTH levels were associated with a low bone mass in the hemodialysis patients. We suggest that regular monitoring of the femoral neck BMD, maintaining an adequate serum albumin level and BW, and undertaking an exercise program are important to improve bone health in the patients undergoing hemodialysis.

## Background

Outcomes of chronic kidney disease (CKD) or chronic renal failure (CRF) include not only progression to end-stage renal disease (ESRD) but also complications of reduced kidney function and increased risk of cardiovascular disease [1]. ESRD requires some modalities of renal replacement therapy such as dialysis or kidney transplantation. The incidence of ESRD in Taiwan increased in the last decade and reached the highest incidence of ESRD in the world [2,3]. The prevalence of ESRD was 1706 per million population in Taiwan, second only to Japan [2]. Nowadays more than 35,000 patients undergo long-term hemodialysis in Taiwan each year [3].

ESRD patients usually have an accelerated bone loss due to abnormal bone turnover that leads to a high prevalence of bone health problems, e.g., osteopenia and osteoporosis etc. [4-11]. Furthermore, parathyroid hormone (PTH)-related bone disease influences bone mineral density (BMD) in hemodialysis patients, in addition to other important risk factors such as advanced age, age at menarche, female gender, and history of previous fractures, etc. On the other hand, protective factors for bone mass loss in this population include body weight (BW), hemoglobin, weekly heparin dose and a history of parathyroidectomy, etc. [7].

ESRD patients with a low bone mass usually have a high incidence of fragility fracture [12,13]. The overall relative risk for hip fracture was 4.4 for dialysis patients compared with general population [12]. It's important to periodically evaluate the BMD in these patients. Nowadays dual-energy x-ray absorptiometry (DXA) is the most recommended method because of its high precision and accuracy, a short scan time and a low radiation dose, in spite of its relatively high cost and radiation modality. DXA is reliable for evaluating the changes of BMD and bone mineral content at different sites [11,14]. Previous studies demonstrated that the amount of bone loss in the femoral neck (FN) is greater than that in the vertebra in ESRD patients [6,9]. Most patients who have a low BMD value in the FN have a higher risk of femoral neck fracture. Thus the FN is the best site for BMD evaluation in dialysis patients. On the other hand, Baszko-Blaszyk et al. [15] suggested DXA measure of forearm BMD as another quick and sensitive way to detect osteoporosis in peritoneal dialysis patients, with FN BMD as a second choice. Yamaguchi [16] advocated the distal radius as the preferred site for bone densitometry in CKD patients. However, Moe et al. [17] stated that the value of bone density measurement in the evaluation of CKD-mineral and bone disorder is not well established. Finding on the correlation of BMD values to fracture risk in the CKD population are inconsistent.

Since the osteoporosis is one of main bone health problems in the CKD patients and the incidence of hemodialysis increased rapidly in Taiwan, this study aims to investigate the status of BMD among hemodialysis patients in Taiwan and to explore the associated factors related to loss of bone mass.

## Methods

This study was a cross-sectional correlation study and was conducted in subjects undergoing hemodialysis at National Taiwan University Hospital (NTUH) from November 2004 to March 2006. This study was the first part of a series of studies, and the second part was a study on the effect of exercise program on BMD of hemodialysis patients. The inclusion and exclusion criteria were defined to fulfill the purposes of these two studies. There were 155 patients on hemodialysis in NTUH during the study period. Patients with an age of 18 years or older from both sexes who have been on hemodialysis three times a week for more than 6 months were included in the study group. Nine patients received hemodialysis twice a week, and 7 patients received hemodialysis less than 6 months, therefore 139 patients were included. The exclusion criteria and the number of excluded patients were as the following: (1) coronary artery disease, 13; (2) orthopedic disorders exacerbated by activity, 10; (3) chronic lung disease resulting in shortness of breath during exercise, 4; (4) a history of cerebrovascular disease, 4; (5) unable to communicate, 3; (6) blindness, 1. Subjects with fracture of the lumbar spine or fracture of right hip were also excluded. There were 104 eligible subjects. The principal investigator invited the eligible subjects to participate the study and 41 subjects declined the request. The reasons included (1) no interest, 19 subjects; (2) no extra time to receive BMD measurement, 12; (3) disagreement by family, 10. Finally 63 hemodialysis patients were recruited into the study. The participation rate was 41% (63/155). Written consent was obtained from each subject. This study was carried out with the approval of the institutional review board of NTUH.

We used a structured interview questionnaire and reviewed medical records of each patient to collect their demographic data and dialysis-related information. Serum albumin, calcium, phosphate and alkaline phosphatase (ALP) were measured every month, and the intact PTH (iPTH) level was measured every 3 months on a regular schedule at this dialysis center. The measurement procedures and reports were validated by the Division of Biochemistry Laboratory at NTUH. Biochemistry data was collected the same month as BMD measurement. The BW was recorded on the day of the biochemistry check before dialysis. The phosphate binders and vitamin D were giving to the patients to keep Ca and PO_4 _balances. No subjects used calcimimetics or steroid during the study period.

Based on the American College of Sports Medicine [18] and Ehrman's studies [19], the intensity of exercise is an important determinant for the efficiency of exercise and heart rate is a good indicator to intensity. In subjects who had regular exercise, the "effective exercise time" was defined as the weekly total exercise time resulting in increased heart rate, respiratory rate, or sweating. For example, if a subject jogged for one hour twice per week with increases in the heart and respiratory rates, the effective exercise time was equal to 120 minutes.

All subjects underwent DXA assay of lumbar spine (LS) and right hip BMD by the same technologist who was certified by the International Society of Clinical Densitometry. The assay was performed using a fan-beam bone densitometer (QDR-4500A; Hologic, Waltham, Massachusetts, USA) [20]. Instrument quality control was performed at least 5 days per week, and the coefficient of variation (CV) for the total BMD was 1.0%. Based on the World Health Organization (WHO) definition, the "normal" BMD is defined as less than 1 standard deviation (SD) below the average BMD of a healthy young adult reference group, i.e. a T-score ≧ -1.0; "osteopenia" as BMD reduced by more than 1 SD but less than 2.5 SD, i.e. a -1.0 > T-score > -2.5; and "osteoporosis" as BMD reduced by more than 2.5 SD, i.e. a T-score ≦ -2.5 [21-23].

All data were statistically analyzed by means of descriptive analysis, independent t test and one way analysis of variance with post hoc test (Scheffe method) for continuous variables. The Pearson product-moment correlation was used to explore correlated factors of BMD, and stepwise multiple linear regression (stepwise forward estimation) was used to identify predictors of low bone mass. Analyses were performed using the SPSS statistical program, version 11.0 for Windows (SPSS Inc., Chicago, IL, USA). All tests were two-sided, and a *p *value < 0.05 was considered statistically significant.

## Results

### Characteristics of subjects

Table 1 shows the demographic data of all 63 subjects. The mean age of the subjects was 55.7 (SD 13.5) years. There were 35 men (56%) and 28 women (44%), of whom 21 were post-menopausal or permanently amenorrheic. There were statistically significant differences in BW, body height, body mass index (BMI), and Kt/V in the gender subgroups (*p *< 0.001, *p *< 0.001, *p *= 0.014, and *p *< 0.001 respectively), but no significant differences in age and hemoglobin by gender. The hemoglobin level was less than normal. Only 1 (3%) male subject and 6 (21%) female subjects had hemoglobin levels in the normal range. The serum ALP in 17 subjects (27%) and iPTH in 54 subjects (86%) were higher than normal and had a wide range. The duration of dialysis averaged 38.8 (SD 9.8, range 6–202) months. Fifty-one subjects (81%) had no previous bone densitometry. Three subjects (5%) had fractures before starting renal replacement therapy, two in the arm and one in left hip treated with a total hip arthroplasty. Six subjects (10%) had undergone parathyroidectomy, and 3 (5%) had renal transplantation. There were no statistically significant differences between these subgroups in the BMD and prevalence of low bone mass at the LS and FN.

**Table 1:** Characteristics and clinical variables of subjects

Characteristic or clinical variable (normal range)	Value	Range	*p *value
			
	mean ± SD or n (%)		
Age (years)	55.7 ± 13.5		*p *= 0.60
Male	56.5 ± 14.4	18–83	
Female	54.7 ± 12.6	27–78	
Body weight (kg)	61.4 ± 11.9		*p *< 0.001
Male	68.1 ± 11.2	49.2–93.4	
Female	53.1 ± 6.1	41.5–66.6	
Body height (cm)	161.4 ± 8.5		*p *< 0.001
Male	166.8 ± 6.4	150.5–182.0	
Female	154.5 ± 5.3	144.0–165.0	
Body mass index (kg/m^2^)	23.5 ± 3.6		*p *= 0.014
Male	24.5 ± 3.9	17.4–34.1	
Female	22.3 ± 2.6	17.9–28.0	
Kt/V	1.5 ± 0.2		*p *< 0.001
Male	1.3 ± 0.2	1.0–1.6	
Female	1.6 ± 0.2	1.2–2.0	
Hemoglobin (g/dl)	10.6 ± 1.2		*p *= 0.85
Male (12.3–18.3 g/dl)	10.6 ± 1.3	7.6–15.4	
Female (11.3–15.3 g/dl)	10.5 ± 1.1	7.5–12.1	
Calcium (8.1–10.4 mg/dl)	9.0 ± 0.8	7.0–11.2	
Phosphate (2.7–4.5 mg/dl)	5.2 ± 1.7	2.7–13.3	
Calcium-phosphate product	46.9 ± 15.2	24.7–97.9	
Albumin (3.5–5.0 g/dl)	4.1 ± 0.3	3.4–4.8	
Alkaline phosphatase (60–220 U/l)	185.4 ± 97.1	72–564	
iPTH (12–72 pg/ml)	264.3 ± 263.7	3.0–1550	
Duration of dialysis (month)	38.8 ± 39.8	6–202	
Effective exercise time (minute/wk)	100.3 ± 206.9	0–1050	
Had bone densitometry history	12 (19%)		
Had fracture history after dialysis	3 (5%)		
Had parathyroidectomy history	6 (10%)		
Had renal transplantation history	3 (5%)		

iPTH, intact parathyroid hormone

### Bone mineral densitometry and prevalence of osteopenia and osteoporosis

The BMD in LS, FN and total hip (TH) sites and prevalence of osteopenia and osteoporosis of these 63 subjects are shown in Tables 2 and 3. The correlation coefficients between BMD values of FN and TH was high (*r *= 0.89, *p *< 0.001). The correlation coefficients between BMD values in LS and FN (*r *= 0.59, *p *< 0.001) and the LS and TH (*r *= 0.72, *p *< 0.001) were lower than those in FN and TH but were still statistically significant. Men had a higher BMD and T-score than women at these three sites. The percentages of subjects with a low bone mass (including osteopenia and osteoporosis) in the LS, FN, TH, and overall were 46%, 73%, 49%, and 81%, respectively. The prevalence rates of osteoporosis at the same sites were 8%, 10%, 5%, and 13%, respectively. Overall, the prevalence rate of osteoporosis in women (21%) was higher than that in men (6%). Of note, two men had osteoporosis at the FN site only. In different age groups, those above 70 years had the highest prevalence of osteoporosis (22%), followed by those 50–59 years (18%).

**Table 2:** Results of bone mass in subjects

Region^a^	Sex	BMD (g/cm^2^)	T-score (range)	Normal^b^ BMD (%)	Osteopenia^b^ prevalence (%)	Osteoporosis^b^ prevalence (%)
LS	All	0.933 ± 0.152	-0.7 ± 1.3 (-3.1~2.1)	54	38	8
	Male	0.979 ± 0.146	-0.4 ± 1.2 (-2.4~2.1)	60	40	0
	Female	0.875 ± 0.140	-1.1 ± 1.2 (-3.1~1.1)	46	36	18
FN	All	0.657 ± 0.099	-1.5 ± 0.9 (-4.0~0.6)	27	63	10
	Male	0.673 ± 0.101	-1.4 ± 0.8 (-2.6~0.6)	26	68	6
	Female	0.637 ± 0.094	-1.5 ± 0.9 (-4.0~-0.3)	29	57	14
TH	All	0.781 ± 0.126	-1.0 ± 0.9 (-3.9~1.1)	51	44	5
	Male	0.827 ± 0.118	-0.8 ± 0.9 (-2.4~1.1)	49	51	0
	Female	0.723 ± 0.114	-1.1 ± 1.0 (-3.9~0.7)	53	36	11
Overall	All	-	-	19	68	13
	Male	-	-	20	74	6
	Female	-	-	18	61	21

Values are presented as means ± SD or percentage; BMD, bone mineral density.

a: Region means the site to take bone mineral densitometry assessment

LS: lumbar spine 1 to 4, FN: femoral neck, TH: total hip, Overall: category defined by the site with the lowest T-score

b: Based on the WHO classification

Normal: T-score ≧ 1.0, Osteopenia:-1.0 > T-score > -2.5, Osteoporosis: T-score ≦ -2.5

**Table 3:** The prevalence of osteoporosis in different age groups of subjects

Age stratum (year)	All					Male					Female				
			
	n	LS	FN	TH	Overall	n	LS	FN	TH	Overall	n	LS	FN	TH	Overall
		n	n	n	n (%)		n	n	n	n (%)		n	N	n	n (%)
< 40	9	0	1	0	1 (11)	4	0	1	0	1 (25)	5	0	0	0	0 (0)
40–49	10	0	0	0	0 (0)	7	0	0	0	0 (0)	3	0	0	0	0 (0)
50–59	17	2	2	1	3 (18)	9	0	1	0	1 (11)	8	2	1	1	2 (25)
60–69	18	1	2	1	2 (11)	9	0	0	0	0 (0)	9	1	2	1	2 (22)
≧ 70	9	2	1	1	2 (22)	6	0	0	0	0 (0)	3	2	1	1	2 (67)

Total	63	5	6	3	8 (13)	35	0	2	0	2 (6)	28	5	4	3	6 (21)

Values are presented as number (percentage)

LS: lumbar spine 1 to 4, FN: femoral neck, TH: total hip, Overall: category defined by the site with the lowest T-score

Based on the WHO classification: Osteoporosis indicates a T-score ≦ -2.5

### Associated factors of BMD

The Pearson's correlation among these nine variable (age, BW, body height, albumin level, ALP level, iPTH level, Kt/V, duration of dialysis, and effective exercise time) were presented in Table 4. The BW was highly correlated to Kt/V (r = -0.69, *p *< 0.001) and ALP to iPTH also had a high correlation (r = 0.54, *p *< 0.001). When independent variables were highly correlated to each other, we should be cautious to use them in the same analysis. The problem of multicollinearity could occur [24], which would make multivariate regression analysis inappropriate. We chose BW and ALP level into the regression model testing because BW and ALP level had higher correlation coefficient to LS BMD than Kt/V and iPTH level. All the independent variables, except body height and duration of dialysis, were significantly correlated with the outcome variable (LS BMD or FN BMD). The age, serum ALP level, serum iPTH level, and Kt/V had negative associations, and the serum albumin level, effective exercise time, and BW had positive associations with LS or FN BMD. For women, BW had a positive association (LS *p *= 0.041, FN *p *= 0.012) with BMD of both sites, but only age had a negative association (*p *< 0.001) with LS BMD. For men, only the serum albumin level had a positive association (*p *= 0.037) with FN BMD, and only the serum iPTH level had a negative association (*p *= 0.020) with LS BMD.

**Table 4:** Correlation between bone mineral density and related variables

Variable	X_1_	X_2_	X_3_	X_4_	X_5_	X_6_	X_7_	X_8_	X_9_	Y_1_	Y_2_
X_1_	1.00										
X_2_	-0.12	1.00									
X_3_	-0.21	0.58***	1.00								
X_4_	0.03	0.08	0.02	1.00							
X_5_	-0.04	0.05	-0.01	-0.20	1.00						
X_6_	-0.19	0.16	0.22	-0.13	0.54***	1.00					
X_7_	0.06	-0.69***	-0.63***	-0.09	0.09	-0.05	1.00				
X_8_	-0.22	-0.28*	-0.17	-0.21	0.17	0.28*	0.20	1.00			
X_9_	0.09	-0.10	0.04	0.07	-0.09	0.04	0.03	-0.13	1.00		
Y_1_	-0.15	0.34**	0.21	0.25*	-0.27*	-0.25*	-0.33**	-0.09	0.12	1.00	
Y_2_	-0.26*	0.21	0.14	0.28*	-0.18	-0.14	-0.22	0.04	0.27*	0.58***	1.00

Date are presented as the r value in Pearson's correlation test

* *p *< 0.05, **p < 0.01, *** *p *< 0.001

X_1_: Age, X_2_: Body weight, X_3_: Body height, X_4_: Albumin, X_5_: Alkaline phosphatase,

X_6_: intact parathyroid hormone, X_7_: Kt/V, X_8_: Duration of dialysis,

X_9_: Effective exercise time,

Y_1_: Lumbar spine BMD, Y_2_: Femoral neck BMD

### Risk factors for reduced BMD

Table 5 shows statistically significant differences in BMD at different sites (LS, FN, or TH) and subgroups. The dependent variables were LS BMD, or FN BMD, or TH BMD. For different sites, every factor was divided into two levels and independent t test was used to define the possible risk factor. However the iPTH was divided into three levels (normal, moderate, and high), and one way ANOVA was used. If the difference was significant, then Post Hoc test (Scheffe's method) was done. There was a significant difference between moderate level and high iPTH level (p = 0.042). For lumbar spine, the subjects who were under 50 years old, men, women before menopause, those in the first year of dialysis, those with an iPTH moderate level in the 1.5- to 5-fold normal range, or those with an albumin ≧ 4.1 g/dl had a significantly higher LS BMD values. For femoral neck, the subjects who were under 50 years old, those with an ALP < 330 U/l (1.5-fold normal range), those with an albumin ≧ 4.1 g/dl, those engaged in impacted exercise, or those had effective exercise for at least 60 minutes per week had a significantly higher FN BMD value. For total hip, the subjects who were men, women before menopause, those with an ALP < 330 U/l, those with an albumin ≧ 4.1 g/dl, or those had effective exercise for at least 60 minutes per week had a significantly higher TH BMD value. Men had a higher bone mass than women, except at FN. Women before menopause had a larger bone mass than those postmenopausal women at LS and TH. There was no association between regular exercise and BMD, but subjects who had effective exercise for at least 60 minutes per week or engaged in impacted exercise had a significantly higher bone mass at FN.

**Table 5:** Comparison of the bone mineral density in different subgroup subjects

Group variable	n	LS BMD	FN BMD	TH BMD
Age (year)		*p *= 0.023 (*)	*p *= 0.019 (*)	*p *= 0.13
< 50	19	0.998 ± 0.140	0.701 ± 0.088	0.818 ± 0.115
≧ 50	44	0.904 ± 0.149	0.638 ± 0.098	0.765 ± 0.128
Gender		*p *= 0.006 (**)	*p *= 0.15	*p *= 0.001(**)
male	35	0.979 ± 0.146	0.673 ± 0.101	0.827 ± 0.118
female	28	0.875 ± 0.140	0.637 ± 0.094	0.723 ± 0.114
Menstrual cycle (n = 28)		*p*<0.001(***)	*p *= 0.10	*p *= 0.014 (*)
yes	7	1.040 ± 0.047	0.687 ± 0.056	0.812 ± 0.085
no	21	0.820 ± 0.114	0.620 ± 0.099	0.693 ± 0.108
Duration of dialysis (month)		*p *= 0.045 (*)	*p *= 0.31	*p *= 0.08
< 13	16	0.998 ± 0.182	0.679 ± 0.123	0.828 ± 0.146
≧ 13	47	0.910 ± 0.135	0.650 ± 0.089	0.765 ± 0.116
iPTH (pg/ml)		*p *= 0.042 (*)	*p *= 0.135	*p *= 0.10
Normal (0–108)	18	0.935 ± 0.174	0.656 ± 0.104	0.790 ± 0.122
Moderate (109–360)	31	0.970 ± 0.138	0.677 ± 0.089	0.804 ± 0.121
High (>360)	14	0.848 ± 0.124	0.613 ± 0.105	0.718 ± 0.130
ALP (U/l)		*p *= 0.10	*p *= 0.019 (*)	*p *= 0.005(**)
< 330	57	0.943 ± 0.151	0.666 ± 0.094	0.795 ± 0.116
≧ 330	6	0.835 ± 0.128	0.568 ± 0.111	0.646 ± 0.153
Albumin(g/dl)		*p *= 0.028 (*)	*p *= 0.032 (*)	*p *= 0.001 (**)
< 4.1	28	0.886 ± 0.138	0.628 ± 0.079	0.727 ± 0.095
≧ 4.1	35	0.970 ± 0.154	0.680 ± 0.107	0.824 ± 0.133
Regular exercise		*p *= 0.22	*p *= 0.54	*p *= 0.96
yes	35	0.911 ± 0.161	0.664 ± 0.106	0.781 ± 0.134
no	28	0.959 ± 0.136	0.648 ± 0.090	0.780 ± 0.117
Exercise type (n = 35)		*p *= 0.39	*p *= 0.028 (*)	*p *= 0.065
impacted	18	0.935 ± 0.152	0.702 ± 0.099	0.822 ± 0.117
un-impacted	17	0.887 ± 0.172	0.624 ± 0.101	0.738 ± 0.142
EE time (min/wk) (n = 35)		*p *= 0.79	*p *= 0.011 (*)	*p *= 0.019 (*)
< 60	13	0.902 ± 0.184	0.606 ± 0.105	0.714 ± 0.146
≧ 60	22	0.917 ± 0.151	0.698 ± 0.093	0.822 ± 0.112

LS: lumbar spine 1 to 4, FN: femoral neck, TH: total hip iPTH: intact parathyroid hormone,

ALP: Alkaline phosphatase, EE: Effective exercise, * *p *< 0.05, ** *p *< 0.01, *** *p *< 0.001

While examining the distribution of the major variables and their skewness indexes, the age, body weight, and serum albumin level were shown to have normal distribution. The iPTH level, ALP level, duration of dialysis, and effective exercise time were positively skewed. The skewness indexes (= skewness/standard error of skewness) were 8.12, 5.64, 7.69, and 9.88, respectively. These four variables were transformed by the suggestions of Tabachinck and Fidell [24]. The square root transformation was applied to transform the iPTH level. The logarithm transformation was applied to transform the ALP level, duration of dialysis, and effective exercise time. After transformation, the skewness indexes for these four variables were 2.73, 1.75, 0.69, and 2.50, respectively. The transformed data were used for regression analysis.

The possible risk factors and biochemical markers were entered as independent variables in a stepwise linear regression analysis. These included age, body weight, serum albumin, alkaline phosphatase, and effective exercise time. The body height and duration of dialysis were excluded because the linear curve-fit test was non-significant. The analysis was used for FN BMD and LS BMD. The scatterplots of residuals of dependent variables (FN BMD or LS BMD) in these two regression models showed a spread out distribution of the residuals against the predicted values. Thus, the assumptions of linearity, homoscedasticity, independence, and normality of residuals were met on the major variables. Table 6 shows that three variables, i.e., age, transformed effective exercise time, serum albumin level, and age significantly predicted the FN BMD; the standardized coefficients (β) were 0.324 (*p *= 0.006), 0.278 (*p *= 0.017), and -0.291 (*p *= 0.013) respectively. These three variables accounted for 25% of the total variance (R^2 ^= 0.25, *p *< 0.01). Another two variables, BW and transformed ALP level, significantly predicted the LS BMD; the standardized coefficients (β) were 0.338 (*p *= 0.005) and -0.285 (*p *= 0.017), respectively. These two variables accounted for 20% of the total variance (R^2 ^= 0.20, *p *< 0.001). No other variable was found to have any significantly predictive power. The standardized coefficient on effective exercise time-log, 0.324, reflected change in the mean FN BMD per 1-unit increase in effective exercise time-log, if albumin level and age stayed the same. Similarly the standardized coefficient on albumin level, 0.278, reflected change in mean FN BMD per 1-unit increase in albumin level, if effective exercise time-log and age stayed the same. The standardized coefficient on age, -0.291, reflected change in the mean FN BMD per 1-unit increase in age, if effective exercise time-log and albumin level stayed the same. The standardized coefficient on BW, 0.338, reflected change in the mean LS BMD per 1-unit increase in BW, if ALP-log stayed the same. Similarly, the standardized coefficient on ALP-log, -0.285, reflected change in the mean LS BMD per 1-unit increase in ALP-log, if BW stayed the same. When the standardized coefficient is high, the influence on FN BMD or LS BMD is high.

**Table 6:** Predicting models of femoral neck BMD and lumbar spine BMD

Model	Standardized coefficients (β)	*P *value	R square	R^2 ^change
Femoral neck BMD			0.25	
Effective exercise time-log	0.324	0.006		0.094
Albumin level	0.278	0.017		0.079
Age	-0.291	0.013		0.077
Lumbar spine BMD			0.20	
Body weight	0.338	0.005		0.118
ALP-log	-0.285	0.017		0.082

BMD: bone mineral density, ALP: Alkaline phosphatase

Effective exercise time-log: transformed effective exercise time = LG_10 _(old value + 1)

ALP-log: transformed ALP = LG_10 _(old value)

## Discussion

Bone mass is a complex trait characterized by multiple determinants, such as gene-environmental interactions [25]. There are multiple factors determining bone mass loss in the CRF patients whose bone quality and bone quantity deteriorate rapidly. Our study found that the percentage of these ESRD subjects with low bone mass (including osteopenia and osteoporosis) at the LS, FN, and TH sites were 46%, 73%, and 49%, respectively. This trend is similar to previous reports [7,9,26]. Although Taal et al. showed that the prevalence of osteoporosis was twice of our findings, both studies revealed FN as the region with the highest prevalence of osteoporosis. Bone loss in renal osteodystrophy was more prominent in the FN than the LS or TH, which is compatible with previous studies [9,26]. We found that the FN was the most likely to show low bone mass, and only 27% of subjects had normal bone mass. Only two men had osteoporosis, both of whom were younger than 60 years old and had osteoporosis at the FN site. Gabay et al. [9] indicated that cortical bone is more sensitive than trabecular bone to CKD-related bone loss in women. Further study is needed to clarify whether the difference is related to characteristics of bone structure (cortical bone or cancellous bone).

The sudden decrease in estrogen level at menopause leads to decreased absorption of calcium, increased osteoclast activity and an accelerated bone loss [4]. In our study, menopause proved to be an important risk factor for decreased BMD in dialysis patients. Yang et al. [27] indicated that there was a trend toward an increasing proportion of coded osteoporosis with age, especially in women, and women in the 50–59 year stratum had a 13-fold higher proportion of osteoporosis than men. Yang et al. [28] surveyed the incidence of osteoporosis in Taiwanese women and found that the incidence in the ≧ 50 years group and total subjects were 11% (391/3582) and 10% (473/4689) in the LS, and 8% (225/2732) and 7% (263/3529) in the FN, respectively. In our study, there was a similar trend in women, but not in men, and women in the 50–59 years stratum only had a 2-fold higher proportion of osteoporosis than men. In women, the incidence of osteoporosis in ≧ 50 years group and total subjects were 25% (5/20) and 18% (5/28) in the LS, and 20% (4/20) and 14% (4/28) in FN. Our sample size was small and the subjects were hemodialysis patients, which may be possible causes for these differences.

Our study showed that one of the three dialysis patients with a history of fracture had osteoporosis and the other two had osteopenia in the LS. This shows that fracture occurs more frequently in patients with low bone mass. One of the three renal transplant patients had osteoporosis and the other two had osteopenia in the FN and two of the three had osteopenia in the LS. The relationship between bone loss and long term use of immunosuppressive agents in transplant patients needs further study.

We found that BMD decreased as age increased and BMD increased as BW increased in LS and FN. There were no associations between body height and BMD at both sites. It was compatible with other previous findings [7,28-30]. In our study, no association was found between the BMD at these two sites and the duration of dialysis, compatible with two other studies [6,31]. The discordance between our results and previous studies may relate to the wide range of duration of dialysis, previous parathyroidectomy or transplantation in some patients in this study. Patients who had dialysis for more than one year had a larger bone loss than those with shorter durations of treatment but this was statistically significant only for the LS BMD. A relationship between bone loss and duration of dialysis could exist, but wasn't detected by our cross-sectional design because of the small sample size, the small quantity of changes occurring in some periods that we still look for. This also indicated that some other factors could not be determined by BMD measurement, such as architecture and bone turnover in different regions of the skeleton.

Our data demonstrated no correlation between BMD values with Ca, PO_4_, and the Ca, PO_4 _, and the Ca × PO_4 _product. Ersoy et al reported that the Ca × PO_4 _product correlated negatively with lumbar T-scores and femoral neck Z-scores in patients receiving peritoneal dialysis [32]. As markers of bone turnover, there were negative associations of ALP and iPTH with LS BMD, but non-linear correlation with FN BMD. Taal et al. [7] showed that the FN BMD had a negative association with iPTH. We found that too high ALP and iPTH levels did not benefit bone mass, but keeping medium high levels of ALP (1.5-fold) and iPTH (1.5- to 5-fold) is helpful for maintaining bone mass. A high iPTH level implicates a more bone resorption, while low to medium iPTH levels implicates a more bone formation [4]. After dialysis, the relationship between iPTH and low bone mass may become more complex and are interfered with many factors. Because bones of CRF patients may show resistance to parathyroid, the iPTH level must have a 2- to 3-fold of the normal range to maintain normal turnover of bone [33]. Kaneko et al. [34] reported that dialysis therapy for more than 3 years carried a higher risk (adjusted hazards ratio, 1.33) and an iPTH value of 227–538 pg/ml, a lower risk (adjusted hazards ratio, 0.68) of long-bone fracture. These results supported our findings, and could be applied in clinical treatment.

Osteopenia is commonly noted in ESRD patients. We need to pay more attention to prevent bone loss in these patients. This study showed that 51 subjects (81%) had low bone mass, and 44 subjects (70%) were more than 50 years old, but only 12 subjects (19%) underwent BMD measurements previously. Monitoring bone mass in dialysis patients is important. Because bone loss is asymptomatic, it is easily neglected. Since the risk of fracture increases with low bone mass, the presence of osteopenia should alert the clinician to pay more attention to their bone health. We need to regularly measure BMD of dialysis patients to find high risk patients to develop fracture, which can lead to mortality and increased medical costs. Three strategies may help reduce the burden of fractures in dialysis patients: (1) Optimize the management mineral metabolism; (2) Apply fall prevention strategies including exercise training; (3) Consider the use of hip protectors in high-risk patients [35].

From the risk factors and predictors of FN and LS BMD in this study, we found that age, BW and the ALP level had important effects. There were similar results shown in other previous studies [7,29,30]. We also found that the serum albumin level and effective exercise times were positively correlated with FN BMD in dialysis patients. The relationship between albumin level and BMD has rarely been reported. Lai et al. [29] showed that there was no association with the serum albumin level and BMD in the hemodialysis patients. Our study revealed that the patients keeping an albumin level over 4.1 g/dl had a higher BMD. Exercise is a non-pharmacologic way of preventing bone loss. The impacted exercise would apply loads to femoral neck, such exercise training leads to increases in bone mass and showed significant effects on FN BMD [36-38]. Our study indicated that regular exercise alone is not a significant factor for preventing bone loss, whereas both exercise type and effective exercise time are important. The intensity of exercise was an important determinant for prevention of involutional bone loss, especially in the ESRD patients. Therefore, designing a rehabilitative exercise program for hemodialysis patients is also an important issue for them. With the modest R^2 ^of 25% and 20% in our study, some other factors affecting BMD need to be defined.

This study has some limitations. It was a single center study with a small patient population and a low participation rate that precluded drawing a firm conclusion from this clinical study because many unmeasured confounders may have played an important role. The BMD survey sites included the FN and LS only, but not the radius. The inclusion and exclusion criteria were defined to fulfill the purposes of the following exercise program study, thus the subjects in this study were relatively healthy. The above-mentioned limitations restrict the generalizablity of our results. This cross-sectional study found some risk factors of low bone mass in the hemodialysis patients. Prospective study may be needed to establish the factors that cause low bone mass. A prospective study of the effects of an exercise program on BMD in hemodialysis patients is ongoing.

## Conclusion

This is a cross-sectional study exploring the prevalence of low BMD and its associated factors in 63 chronic hemodialysis patients. We found a high percentage of reduced bone mass in these subjects, and the most common region of bone loss was the femoral neck. Advanced age, low BW, high ALP level, and high iPTH level about 5 folds of normal upper limit had adverse effects on maintaining bone mass. Since bone loss is an asymptomatic process, early identification of small reductions in bone mass is very important to allow early intervention to prevent bone loss. To assure the quality bone care, we need to monitor the BMD of femoral neck regularly, to maintain a satisfactory serum albumin level, as well as to promote an exercise program.

## Competing interests

The authors declare that they have no competing interests.

## Authors' contributions

GSH, TSC, SLH, and RSY were responsible for the design of this study. MFL provided analytical support. GSH, TSC, and RSY prepared the writing of the manuscript. All authors read and approved the final manuscript.

## Pre-publication history

The pre-publication history for this paper can be accessed here:



## References

[B1] Levey AS, Atkins R, Coresh J, Cohen EP, Collins AJ, Eckardt KU, Nahas ME, Jaber BL, Jadoul M, Levin A, Powe NR, Rossert J, Wheeler DC, Lameire N, Eknoyan G (2007). Chronic kidney disease as a global public health problem: Approaches and initiatives – a position statement from Kidney Disease Improving Global Outcomes. Kidney Int.

[B2] National Kidney Foundation (2007). United States Renal Data System 2006 annual data report: International comparisons. Am J Kidney Dis.

[B3] Taiwan Society of Nephrology 2004 Annual report of dialysis patients. http://www.tsn.org.tw/UI/H/H002.aspx.

[B4] Lindberg JS, Moe SM (1999). Osteoporosis in end-stage renal disease. Semin Nephrol.

[B5] Chan TM, Pun KK, Cheng IK (1992). Total and regional bone densities in dialysis patients. Nephrol Dial Transplant.

[B6] Stein MS, Packham DK, Ebeling PR, Wark JD, Becker GJ (1996). Prevalence and risk factors for osteopenia in dialysis patients. Am J Kidney Dis.

[B7] Taal MW, Masud T, Green D, Cassidy MJD (1999). Risk factors for reduced bone density in haemodialysis patients. Nephrol Dial Transplant.

[B8] Barnas U, Schmidt A, Seidl G, Kaider A, Pietschmann P, Mayer G (2001). A comparison of quantitative computed tomography and dual x-ray absorptiometry for evaluation of bone mineral density in patients on chronic hemodialysis. Am J Kidney Dis.

[B9] Gabay C, Ruedin P, Slosman D, Bonjour JP, Leski M, Rizzoli R (1993). Bone mineral density in patients with end-stage renal failure. Am J Nephrol.

[B10] Gerakis A, Hadjidakis D, Kokkinakis E, Apostolou T, Raptis S, Billis A (2000). Correlation of bone mineral density with the histological findings of renal osteodystrophy in patients on hemodialysis. J Nephrol.

[B11] Gal-Moscovici A, Sprague SM (2007). Osteoporosis and chronic kidney disease. Semin Dial.

[B12] Alem AM, Sherrard DJ, Gillen DL, Weiss NS, Beresford SA, Heckbert SR, Wong C, Stehman-Breen C (2000). Increased risk of hip fracture among patients with end-stage renal disease. Kidney Int.

[B13] Stehman-Breen CO, Sherrard DJ, Alem AM, Gillen DL, Heckbert SR, Wong CS, Ball A, Weiss NS (2000). Risk factors for hip fracture among patients with end-stage renal disease. Kidney Int.

[B14] DeVita MV, Rasenas LL, Bansal M, Gleim GW, Zabetakis PM, Gardenswartz MH, Michelis MF (1992). Assessment of renal osteodystrophy in hemodialysis patients. Medicine.

[B15] Baszko-Blaszyk D, Grzegorzewska AE, Horst-Sikorska W, Sowinski J (2001). Bone mass in chronic renal insufficiency patients treated with continuous ambulatory peritoneal dialysis. Adv Perit Dial.

[B16] Yamaguchi T, Kanno E, Tsubota J, Shiomi T, Nakai M, Hattoti S (1996). Retrospective study on the usefulness of radius and lumbar bone density in the separation of hemodialysis patients with fractures from those without fractures. Bone.

[B17] Moe S, Drueke T, Cunningham J, Goodman W, Martin K, Olgaard K, Ott S, Sprague S, Lameire N, Eknoyan G (2006). Definition, evaluation, and classification of renal osteodystrophy: a position statement from kidney disease: improving global outcomes (KDIGO). Kidney Int.

[B18] American College of Sports Medicine (2000). ACSM's Guidelines for Exercise Testing and Prescription.

[B19] Ehrman JK, Ehrman JK, Gordon PM, Visich PS, Keteyian SJ (2003). General exercise prescription development. Clinical Exercise Physiology.

[B20] Shin TTF, Liu HC, Chang CJ, Wei SY, Shen LC, Yang PC (2004). Correlation of MR lumbar spine bone marrow perfusion with bone mineral density in female subjects. Radiology.

[B21] Arden N, Cooper C, Meunier PJ (1998). Present and future of osteoporosis: epidemiology. Osteoporosis: Diagnosis and Management.

[B22] Cunningham J, Sprague SM, Cannata-Andia J, Coco M, Cohen-Solal M, Fitzpatrick L, Goltzmann D, Lafage-Proust MH, Leonard M, Ott S, Rodriguez M, Stehman-Breen C, Stern P, Weisinger J, Osteoporosis Work Group (2004). Osteoporosis in chronic kidney disease. Am J Kidney Dis.

[B23] Wolf RL, Penrod J, Cauley JA, Gueldner SH, Burke MS, Smiciklas-Wright H (2000). Epidemiology: the magnitude of concern. Preventing and Managing Osteoporosis.

[B24] Tabachinck GB, Fidel LS (1996). Using Multivariate Statistics.

[B25] Rosen CJ, Avioli LV (2000). The genetics of osteoporosis. The Osteoporotic Syndrome: Detection, Prevention, and Treatment.

[B26] Rix M, Andreassen H, Eskildsen P, Langdahl B, Olgaard K (1999). Bone mineral density and biochemical markers of bone turnover in patients with predialysis chronic renal failure. Kidney Int.

[B27] Yang NP, Deng CY, Chou YJ, Chen PQ, Lin CH, Chou P, Chang HJ (2006). Estimated prevalence of osteoporosis from a nationwide health insurance database in Taiwan. Health Policy.

[B28] Yang TS, Chen YR, Chen YJ, Chang CY, Ng HT (2004). Osteoporosis: prevalence in Taiwanese women. Osteoporos Int.

[B29] Lai MN, Hsu IS, Chen YY, Gee MJ, Kao MT (2002). Osteoporosis and associated risk factors for chronic hemodialysis patients. Acta Nephrol.

[B30] Ota S, Takahashi K, Taniai K, Makino H (1997). Bone metabolism and daily physical activity in women undergoing hemodialysis (in Japanese). Nippon Jinzo Gakkai Shi (Jpn J Nephrol).

[B31] Spindler A, Paz S, Berman A, Lucero E, Contino N, Penalba A, Tirado S, Santana M, Zeballos AC (1997). Muscular strength and bone mineral density in haemodialysis patients. Nephrol Dial Transplant.

[B32] Ersoy FF, Passadakis SP, Tam P, Memmos ED, Katopodis PK, Özener C, Akcicek F, Cumsari T, Ates K, Ataman R, Vlachojannis JG, Dombros AN, Utas C, Akpolat T, Bozfakioglu S, Wu G, Karayaylali I, Arinsoy T, Stathakis PC, Yavuz Mahmut, Tsakiris JD, Dimitriades CA, Yilmaz ME, Giiltekin M, Karayalcin B, Yardimsever ME, Oreopoulos DG (2006). Bone mineral density and its correlation with clinical and laboratory factors in chronic peritoneal dialysis patients. J Bone Miner Metab.

[B33] Goodman WG (2001). Recent development in the management of renal osteodystrophy. Kidney Int.

[B34] Kaneko TM, Foley RN, Gilbertson DT, Collins AJ (2007). Clinical epidemiology of long-bone fractures in patients receiving hemodialysis. Clin Orthop Relat Res.

[B35] Jadoul M (2007). Towards the prevention of bone fractures in dialysed patients?. Nephrol Dial Transplant.

[B36] American College of Sports Medicine (1995). ACSM position stand on osteoporosis and exercise. Med Sci Sports Exer.

[B37] French SA, Fulkerson JA, Story M (2000). Increasing weight-bearing physical activity and calcium intake for bone mass growth in children and adolescents: a review of intervention trials. Prev Med.

[B38] Heinonen A, Kannus P, Sievanen H, Oja P, Pasanen M, Rinne M, Uusi-Rasi K, Vuori I (1996). Randomised controlled trial of effect of high-impact exercise on selected risk factors for osteoporotic fractures. Lancet 1996 Nov 16.

